# PSICalc: a novel approach to identifying and ranking critical non-proximal interdependencies within the overall protein structure

**DOI:** 10.1093/bioadv/vbac058

**Published:** 2022-08-18

**Authors:** Thomas D Townsley, James T Wilson, Harrison Akers, Timothy Bryant, Salvador Cordova, T L Wallace, Kirk K Durston, Joseph E Deweese

**Affiliations:** Department of Computational Sciences, College of Computing & Technology, Lipscomb University, Nashville, TN 37204, USA; Department of Pharmaceutical Sciences, College of Pharmacy and Health Sciences, Lipscomb University, Nashville, TN 37204, USA; Department of Pharmaceutical Sciences, College of Pharmacy and Health Sciences, Lipscomb University, Nashville, TN 37204, USA; Department of Pharmaceutical Sciences, College of Pharmacy and Health Sciences, Lipscomb University, Nashville, TN 37204, USA; Department of Pharmaceutical Sciences, College of Pharmacy and Health Sciences, Lipscomb University, Nashville, TN 37204, USA; FMS Foundation, Canandaigua, NY 14424, USA; Department of Computational Sciences, College of Computing & Technology, Lipscomb University, Nashville, TN 37204, USA; School of Applied Computational Sciences, Department of Biomedical Data Science, Meharry Medical College, Nashville, TN 37208, USA; Department of Research and Publications, Digital Strategies, Langley, BC V2Y 1N5, Canada; Department of Pharmaceutical Sciences, College of Pharmacy and Health Sciences, Lipscomb University, Nashville, TN 37204, USA; Department of Biological, Physical, and Human Sciences, Freed-Hardeman University, Henderson, TN 38340, USA; Department of Biochemistry, Vanderbilt University School of Medicine, Nashville, TN 37232, USA

## Abstract

**Motivation:**

AlphaFold has been a major advance in predicting protein structure, but still leaves the problem of determining which sub-molecular components of a protein are essential for it to carry out its function within the cell. Direct coupling analysis predicts two- and three-amino acid contacts, but there may be essential interdependencies that are not proximal within the 3D structure. The problem to be addressed is to design a computational method that locates and ranks essential non-proximal interdependencies within a protein involving five or more amino acids, using large, multiple sequence alignments (MSAs) for both globular and intrinsically unstructured proteins.

**Results:**

We developed PSICalc (Protein Subdomain Interdependency Calculator), a laptop-friendly, pattern-discovery, bioinformatics software tool that analyzes large MSAs for both structured and unstructured proteins, locates both proximal and non-proximal inter-dependent sites, and clusters them into pairwise (second order), third-order and higher-order clusters using a k-modes approach, and provides ranked results within minutes. To aid in visualizing these interdependencies, we developed a graphical user interface that displays these subdomain relationships as a polytree graph. To demonstrate, we provide examples of both proximal and non-proximal interdependencies documented for eukaryotic topoisomerase II including between the unstructured C-terminal domain and the N-terminal domain.

**Availability and implementation:**

https://github.com/jdeweeselab/psicalc-package

**Supplementary information:**

Supplementary data are available at *Bioinformatics Advances* online.

## 1 Introduction

A major step forward in predicting the three-dimensional structure of globular proteins was demonstrated at CASP14 with the introduction of a completely redesigned version of AlphaFold (Jumper *et al.*, 2021). Predicting or solving the structure of a protein, however, still leaves the problem of determining which interdependent sites within the primary sequence are essential to produce a functional protein. Highly conserved sites are obviously important, but there may be higher-order interdependencies involving two, three, four or more sites that may be essential to either overall structural stability or function. Direct coupling analysis (DCA) is useful for predicting two- and three-site relationships that are proximal after folding (Schmidt and Hamacher, 2018). However, proximal interactions are not all equally essential, and there are essential interactions that may not be proximal. Binding, for example, may involve two or more sites within the primary sequence that are not proximal in the folded structure, but essential for functionality. Furthermore, there are many intrinsically unstructured proteins that carry out essential functions that are likely to have components that are functionally interdependent and not proximal (Dyson and Wright, 2005; van der Lee *et al.*, 2014). Statistical coupling analysis (SCA) can aid in the discovery of non-proximal relationships but is limited to pairwise correlations (Rivoire *et al.*, 2016). The design of effective synthetic proteins or mRNA and protein sequences can greatly benefit from a tool, therefore, that picks up where AlphaFold, DCA and SCA leave off.

Inference of structural relationships from an amino-acid sequence provides important context to the understanding of a protein across observed sequences. Thanks to more recent advances in high-throughput sequencing technology, the number of available sequences of the same protein across different species has led to great interest in the study of conservation and variation in multiple sequence alignments (MSAs). Even for solved structures, there is still the problem of understanding the substructural relationships between components across the global contact map (Durston *et al.*, 2012; Sulkowska *et al.*, 2012).

Durston *et al.* (2012) applied a derivation of the k-modes algorithm to protein families to discover interdependencies between MSA columns, where each column represents an individual site in the amino acid sequence, but more broadly contains a general description of that site across all sequences. This approach successfully identified not only pairwise dependencies within the protein structure, but much larger mutual interdependencies within a nested hierarchy of clusters, revealing key areas in the 3D structure that were highly interdependent. The nested hierarchy of clusters represented in the cluster tree also showed the branches and where they merged, revealing the final stage of folding for ubiquitin, thus shedding light on the relationship between sequence interdependencies and protein folding.

The approach Durston *et al.* used, however, was inadequate for larger proteins and MSA’s, requiring several individual, decoupled processing steps. The computational complexity made it impractical to work with alignments much larger than 100 amino acids. Our goal in this work is to develop a novel method combining two different concepts, mutual information and k-modes clustering, in a two-phase approach, to analyze large MSAs for proteins of any size, and to discover the key functional and structural sub-molecular components of both globular and intrinsically disordered proteins. This should reveal interdependencies within proteins that may not be discoverable via DCA, as well as predict and rank the most critical components of a protein. To that end, we developed *PSICalc* (Protein Subspace Interdependency Calculator) as a Python-based software tool along with *PSICalc Viewer*, which enables researchers to visualize hierarchical subdomain relationships as polytree graphs. The following will describe our approach and details of the implementation followed by examples of the application to specific protein families with a particular focus on topoisomerase II (TOP2).

## 2 Methods

### 2.1 Application of *PSICalc* to multiple sequence alignments

Within an MSA, we treat an aligned site as a column of amino acids at a given position. If aligned sites within an MSA share a high amount of mutual information, those sites must have a strong interdependency, corresponding to either proximity in the 3D structure and/or a structural or functional relationship (Cofré *et al.*, 2019; Durston *et al.*, 2012). For example, residues may be part of a larger fold or binding site, though they may not be in close proximity in the 3D structure. Thus, intra-group interdependencies in a sequence may not even require geometric proximity among amino acid positions in the MSA to display important interdependent associations between residues.

### 2.2 Multiple sequence alignments as a data structure

There are a few steps required to properly transform MSAs and their corresponding columnar elements into a data structure fit for running the *PSICalc* algorithm. Below we provide formalizations of the data transformation techniques used at each step—staying agnostic to the underlying technology involved so that implementing the *PSICalc* approach in other languages may not be unnecessarily cumbersome. For Python-specific details on the algorithm, please visit the open-source link available on GitHub (https://github.com/jdeweeselab/psicalc-package). All data transformation functions are well-annotated and describe the process within the code.

The process begins with a FASTA-aligned MSA uploaded as either a FASTA or comma-separated values file. First, the MSA is loaded into memory as an M×N matrix where *N* is the number of aligned attribute sites (columns) and *M* is the number of sequences in the alignment (rows). We retain sequence labels identifying the name and labeling schema for later use.

As a final analog to the underlying sequence data, each column must have a unique integer identifier based on the location in the sequence. Throughout the work, we refer to columns and their associated numeric labels as *attributes*. For more information, see the Supplementary Sections S1–S3.

### 2.3 *K*-modes clustering and normalized mutual information

*k-*modes clustering is an unsupervised clustering method that groups attributes about a mode. The *mode* of a cluster is the attribute that has the highest total mutual information with the other attributes forming the cluster. The mode(s) must be discovered by calculating the mutual information between each attribute and all the remaining attributes, starting with pairwise relationships, then increasing cluster size and re-calculating the modes for each cluster. Our methodology is underpinned by the *k-*modes clustering algorithm (Huang, 1997, 1998; Leisch, 2006; Maechler *et al.*, 2019; Müllner, 2013; Nieweglowski, 2020; Suzuki and Shimodaira, 2019) and is explained in Supplementary Section S5.

### 2.4 Deriving mutual information from Shannon uncertainty

Assuming two attributes, each representing a column in an MSA, the mutual information between a given amino acid in one column and those associated with it in the other column, is defined by the probability of the amino acids in the other columns being associated with the given amino acid in the first column. The total mutual information between the two columns is the sum of the mutual information associated with each amino acid. For a detailed mathematical definition, please see Supplementary Section S4.

The total mutual information for each attribute can be normalized to produce a measure of strength between 0 and 1, with 1 being perfectly similar and 0 being mutually exclusive. The closer to 1 the score, the more mutual information and thus the higher certainty the sites share a relationship while a score closer to 0 indicates there is little to no relationship. Note that the score is also symmetric so that the normalized mutual information (NMI) is the same regardless of which attribute is used as the reference attribute. The *PSICalc* implementation is also agnostic to permutations in the two attributes, so that any rearrangements of amino acids within either attribute will not affect the score. In other words, sequence ordering in the MSA does not matter and will not affect the outcome.

### 2.5 Higher order relationships

Estimating interdependence (*SR*) of an attribute *A* with other attributes in the cluster involves summing the NMI for all sets of (*i, i′*) attribute pairs. For example, for a cluster of five attributes, there will be a total *Q* of four attribute pairs associated with a given attribute in the cluster. Let the statistical redundancy (*SR*) be defined as

(1) $$SR\left(i\right)=\sum_{\forall \left(i,i\prime \right)\in Q}\mathrm{NMI}\left(i,i\prime \right)$$

where *i* is the *i*-th amino acid column attribute in the sequence and *Q* is the set of (*i, i′*) attribute pairs (Durston *et al.*, 2012; Wong *et al.*, 1976). For further discussion, see Supplementary Section S4.

### 2.6 Evaluating the overall strength of a cluster

*SR*(*i*) provides insight into which attribute within a cluster contains the strongest location for observing patterns of mutual information. Therefore, the attribute with the highest *SR* value is designated as the *mode* of the cluster. Let the attribute labeled j* denote the mode for the *i*-th subset of the *k*-cluster set as follows:

(2) $$j^{*}=\arg\underset{j\in s_{i}}{\max}\left\{SR\left(j\right)\right\}$$

where {*s*_1_, *s*_2_, …, *s_i_*, …, *s_k_*} = *Q_k_* is the set of clustering memberships subsets.

During execution of the algorithm, clusters select single attributes to merge based on the mode of that cluster as if the cluster represented a single attribute (i.e. the mode). The designated mode of a cluster therefore is always what is measured by another cluster, not the cluster itself, meaning clusters are always measuring the strongest attributes of each other rather than *all* of their attributes. To quantify the overall strength of the cluster when comparing to other clusters in the global map, the designated mode’s *SR* value must be normalized by dividing it by the number of attribute pairs in the cluster set *Q*. Let the statistical redundancy mode (SRM) value of the cluster be defined as:

(3) $$\mathrm{SRM}\left(j^{*}\right)=SR\left(j^{*}\right)/\left|Q\right|$$

where |Q| is the cardinality of the set Q.

The practical implication of the SRM is that it allows for the comparison of two clusters with different numbers of attributes within them and enables us to identify and rank the sub-molecular interdependencies that are the strongest and, therefore, likely the most important within a protein structure.

This method combines a two-phase approach. The first phase discovers the strongest pairwise interdependencies between columns of the MSA according to their mutual information values. In the second phase, *k-*modes clustering is used to cluster the pairwise clusters and the remaining single attributes, into higher third, fourth, fifth order, and higher clusters and output these associations as well as produce a nested hierarchical cluster tree for the protein so that the researcher can observe how the clusters are related to the overall protein function and structure.

## 3 Results

*PSICalc* reveals interdependencies that, according to their SRM values, should be very significant for structure and/or function. Since it can detect non-proximal associations that are not discoverable by DCA, it is important to verify that they are meaningful. When a statistical analysis of *PSICalc* results is conducted, it shows that the discovered interdependencies are highly statistically significant, with *P*-values much <0.0001.

**Fig. 1.**
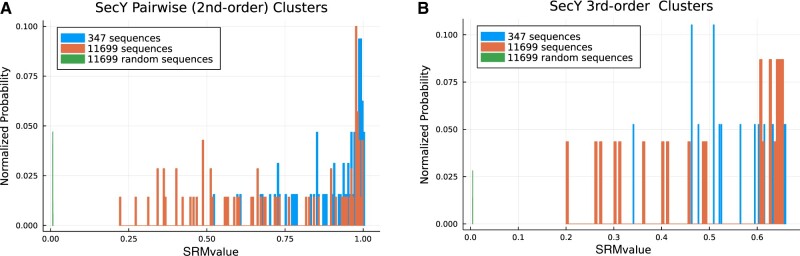
SecY versus random MSA analysis of pairwise and third-order clusters. (**A**) Normalized probability distribution of pairwise cluster SRM values from SecY and random sequences. (**B**) Normalized probability distribution of third-order cluster SRM values from SecY and random sequences

For example, Figure 1 shows a statistical analysis of the universal protein family SecY with pairwise (second order) results in the upper graph and third-order results in the lower. The results in terms of normalized probability versus SRM value are plotted for three data sets: a full set of 11 699 unique SecY sequences (red), a subset of 347 SecY sequences with high similarity (blue) and a set of 11 699 random sequences (green). The random sequences represent the null hypothesis where there are no predicted associations with any significant SRM value.

For the full 11 699 set of unique sequences, the discovered second-order cluster distribution had a mean SRM value of 0.72 (95% confidence interval: 0.66–0.77). This represents a very large deviation from the null hypothesis distribution (green), with a two-sided *P*-value of 10^−35^ and a *t*-statistic of 24.7. For the discovered third-order cluster distribution, the mean value was 0.50 (95% confidence interval: 0.43–0.57). This represents a deviation from the null hypothesis with a two-sided *P*-value of 10^−12^ and a *t*-statistic of 15.5. A similar analysis for the smaller set of 347 unique sequences, resulted in deviations from the null hypothesis with two-sided *P*-values of 10^−56^ and 10^−15^, respectively. A statistical analysis of PSICalc results shows that the discovered interdependent clusters are highly significant. The statistical significance of higher order clusters, such as fourth, fifth, sixth and so on, can be verified using this same method.

### 3.1 Experimental results

To test *PSICalc*, we ran the algorithm on over 35 protein family datasets aggregating top results from phase I sample spreads of 2–7. As seen in Supplementary Table S1, the top clusters from second, third, fourth and fifth order groupings are reported. Protein families included globular, fibrous and intrinsically disordered proteins. The results demonstrate that interdependencies with significant SRM values are identified across various protein family types.

*PSICalc* was also used to examine an alignment of 347 sequences of topoisomerase IIα (TOP2A) across dozens of species gathered from a BLAST search using human TOP2A as the query. To make site mapping simpler, the results were numbered using the human TOP2A as the template to track locations of clustered sites.

**Fig. 2.**
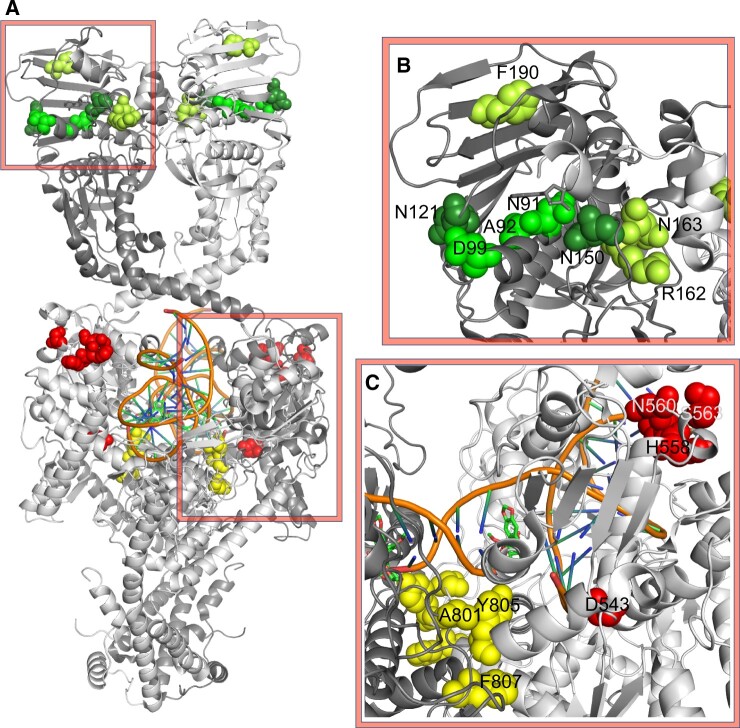
(**A**) TOP2A homodimer with monomers in light grey and dark grey. Examples of clustered residues shown as spheres. (**B**) ATPase domain key residues are anotated around the ATP binding pocket in groupings (N91/A92/D99, green; N121/N160, dark green; R162/N163/F190, light green). (**C**) DNA cleavage/ligation core domain key residue examples from the TOPRIM (red, upper right) and active site (yellow, lower left) are shown as spheres. Images generated from PDB 6ZY7 using Pymol

As seen in Figure 2, *PSICalc* identified several key catalytic clusters that represent physically proximal interactions. Key sites in the ATPase domain were identified including the following groupings (shown as different shades of green): N91, A92, D99 (ATP binding pocket); N121, G122, N150 (also around ATP binding pocket); and R162, N163, F190 (ATP ‘lid’ region).

*PSICalc* also discovered clusters in the cleavage/ligation domain. For example, key residues of the TOPRIM metal binding domain (D543, H558, H559, N560, S563, yellow) and active site (A801, Y805, F807, red) were identified. Additional structurally proximal clusters were also identified by *PSICalc*.

Not only do structurally proximal, key catalytic sites cluster together, *PSICalc* also identified nonproximal interdomain interdependencies. In Supplementary Figure S5, several examples of these interdependencies are mapped using color coding onto the dimer of TOP2A. While these sites are distant in the three-dimensional structure, several of them represent important sites or are in key regions involved in catalysis, interdomain coordination, or domain movements. These interdependencies may help clarify how long-range communication and coordination is occurring in topoisomerase II enzymes. For example, some of the residues in Supplementary Figure S5 and Table S2 interact with DNA and may facilitate domain coordination during binding and cleavage.

**Fig. 3.**
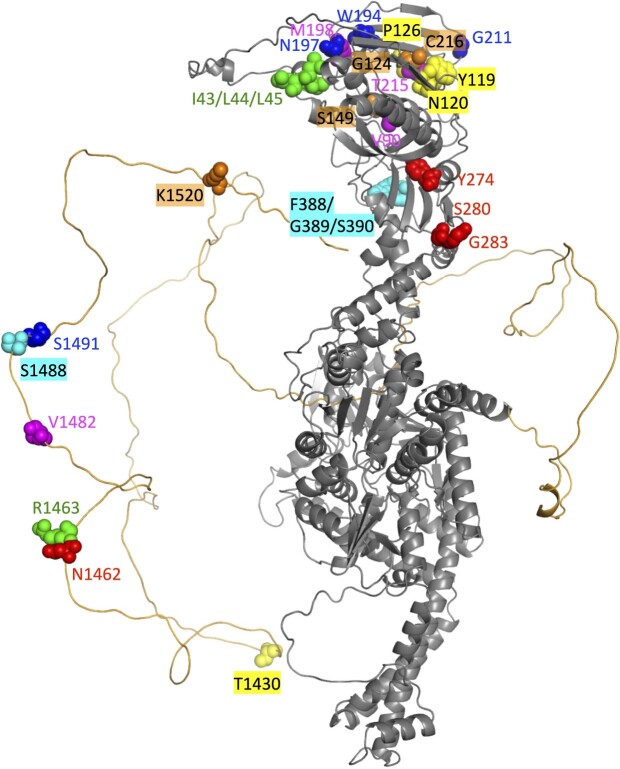
TOP2A monomer is shown with color-coded clusters mapped onto the structure. C-terminal domain is highlighted in orange (long, unstructured domain). Image generated from AlphaFold P11388-F1-model using Pymol

While modern folding algorithms like AlphaFold can solve complex 3D protein structures, there remains the challenge of studying unstructured protein domains. In Figure 3, the AlphaFold structure of a monomer of human TOP2A is shown with the unstructured C-terminal Domain colored orange wrapping around the folded monomer (grey). *PSICalc* discovers multiple interdependencies between sites along the C-terminal domain and the ATPase domain (color-coded residues on structure). While these relationships have not been reported in the literature, we must not ignore the fact that the high SRM values clearly indicate interdependencies. The motivation for the development of this PSICalc approach is to discover and rank strong interdependencies that we have not previously had the tools to detect. Thus, such results can point to new areas of research within a given protein or domain. We propose that these results represent a key ability of this algorithm to identify interdependencies that reflect important functional relationships.

As evidence that these clusters are meaningful, recent work by our lab supports the role of the C-terminal domain in regulating enzyme activity, and multiple interdependencies are consistent with those results (Dougherty *et al.*, 2021). For example, mutation of T1430 (yellow) along with some neighboring residues led to a decrease catalytic activity, which suggests an interaction with the ATPase domain (Dougherty *et al.*, 2021). Importantly, Y119, N120 and P126 line the ATP binding pocket in the ATPase domain. Additionally, mutation of S1488 (cyan) and S1491 (blue) led to an increase in catalytic activity, again supporting the notion that this region may also be interacting with the ATPase domain (W194, N197, G211, blue) and the transducer region (F388, G389, S390, cyan) (Dougherty *et al.*, 2021). Additional sites were identified in the clustering data that were either mutated or neighbor sites mutated in our previous study and coincide with changes in enzyme activity (e.g. T1327, T1470, D1472).

Further studies are underway to combine the cluster results with biochemical assays using point mutations to validate and further explore these interdependencies (J. E. Deweese, unpublished results). Together, this suggests that *PSICalc* may supplement current structure prediction methods by identifying non-proximal relationships even between unstructured and structured domains.

**Fig. 4.**
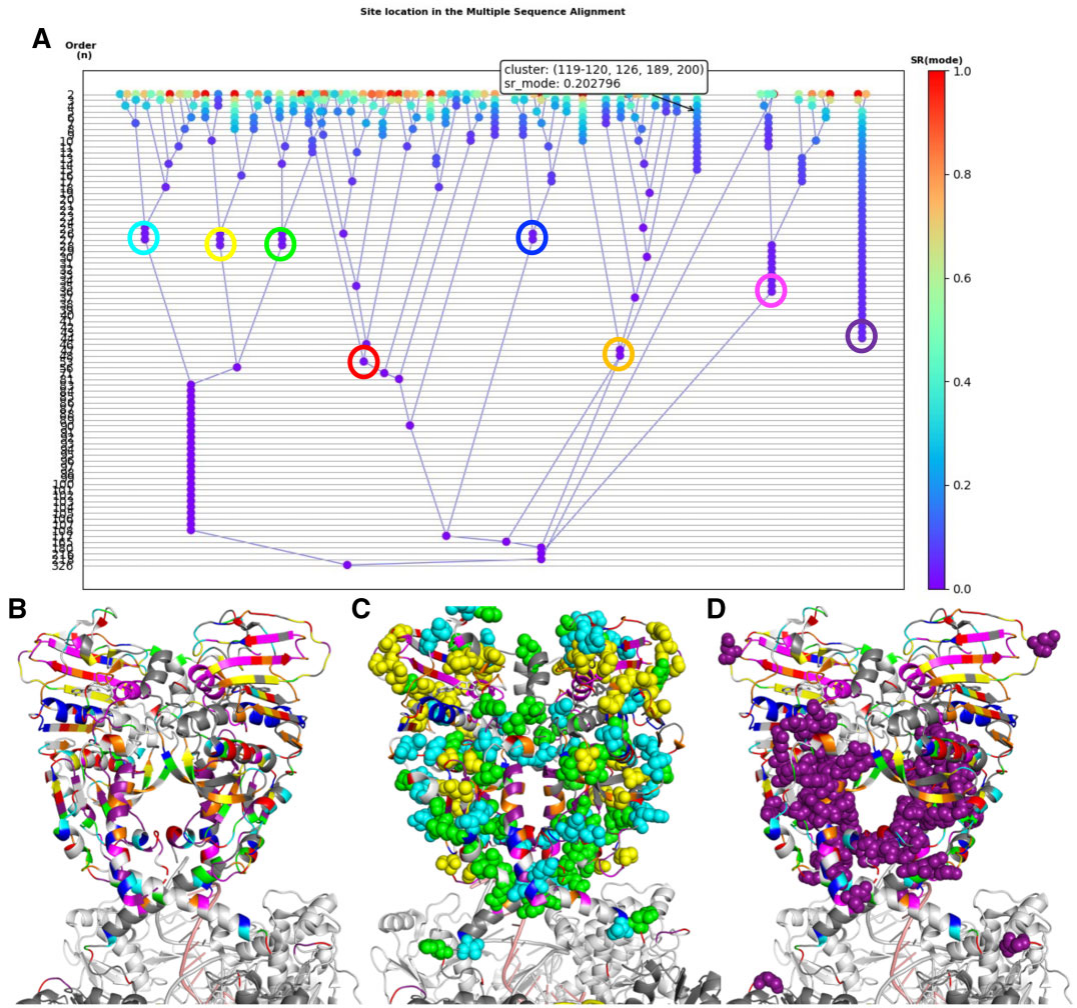
Cluster mapping using *PSICalc Viewer* graph of TOP2A ATPase domain data. (**A**) Polygraph tree of the ATPase domain data from TOP2A. In the GUI version, hovering over a point will provide information about the amino acid position numbers in a cluster (given in parentheses). Color coding of the nodes indicate the SRM value [SR(Mode)] as shown by the scale at the right. Clusters from the polygraph tree can be mapped on to structures using Pymol. (**B**) All circled clusters in (A) mapped to amino acids in ribbon diagram form; (**C**) first three left-most clusters in (A) [yellow, green and cyan] mapped as spheres; (**D**) left-most cluster in (A) [purple] mapped as spheres. Colors correspond to circled clusters in A. Note that the left-most cluster in (A) represents a cluster that does not group in with the remaining clusters. The cluster is an inner core region of the transducer domain, which is involved in communication between the ATPase domain and the rest of the protein. In the GUI version, hovering over a point provides information about the amino acid position numbers in a cluster (given in parentheses). Results represent a spread of 1 with 35% cutoff mapped to the human TOP2A ATPase domain (residues 1–450 out of the full protein). Images generated from PDB 6ZY7 using Pymol. PDB 6ZY7 does not have structural data for the first 28 amino acids since it is unstructured, so these have not been mapped

The PSICalc Viewer also features a polygraph tree for data visualization (Fig. 4). This interactive feature allows researchers to find relationships between sites and examine how groupings fold into larger clusters. Clusters are heat-mapped to SRM values and stratified by cluster order. Connecting lines help trace the combining of interdependent clusters. As seen in Figure 4, clusters can be mapped onto structure using Pymol or similar applications. In the case of the ATPase domain of TOP2A, these clusters correlate with regions involved in domain movement and coordination during the catalytic cycle. However, it must be noted that as the cluster grows larger confidence level decreases. Thus, conclusions based upon larger clusters must be held tentatively until examined with structural and/or biochemical evidence.

## 4 Discussion

Using a derivation of the k-modes algorithm, we have developed a software tool that discovers the most critical non-proximal, as well as, proximal, interdependencies in protein structure. In developing the tool, several key issues were addressed: first, we utilized an MSA matrix definition to store the data and encoded the sequences using numerical substitution. Second, we utilized the contingency matrix nullification approach to address the issue of gaps in the sequence, while still extracting meaningful information from columns with gaps. Third, we developed a way to normalize the data to avoid exaggerated data derived from a standardized normalization approach. While some of these interdependencies represent proximal bonding and Van der Waals interactions others represent distant fold and domain-level associations.

Special consideration should be given to normalization in future and/or derived works. *PSICalc* handles cases where two columns are perfectly invariant i.e. neither column contains more than one symbol from the state space, in which case they are scored by the algorithm as being highly important. However, MSAs in practice have contained ‘imperfectly invariant’ columns where a column contains more than one symbol from the state space, but the vast majority is one symbol. This leads to very low entropy calculations, which in many cases is not a large enough value to normalize the obtained mutual information between 0 and 1. As in the original *k*-modes work, these multi-symbol invariant sites are typically not usable due to a failure to normalize. The importance of invariant positions in the 3D structure is likely very high given the lack of variation at a given site. Therefore, these positions do not need to be ignored in terms of the overall protein and structure. However, this method may be used to supplement other approaches. A future statistical approach to identifying and flagging imperfectly invariant columns could increase *PSICalc’s* ability to detect important invariant relationships.

The *PSICalc* tool enables the discovery of key clusters within protein domains, between domains, and even between intrinsically disordered and ordered domains. As seen in Supplementary Table S1, the software tool can be applied across a range of different protein families representing various types of protein structures including globular, fibrous and intrinsically disordered proteins or protein domains. It is clear from the TOP2A data that interdependencies can include physical proximity, but longer-range relationships are also discovered. We expect that some of these non-proximal interdependencies represent key sites within the structure and may be critical for folding or function. Indeed, as Durston *et al.* (2012) point out, folding domains of ubiquitin can be predicted using this approach.

Importantly, the TOP2A interdependencies between the unstructured C-terminal domain and the ATPase domain represent a key breakthrough that may enable the discovery of a new generation of drugs able to target TOP2A via the C-terminal domain (Murphy *et al.*, 2017). In the case of TOP2A, targeting of the C-terminus would enable selective targeting of TOP2A while avoiding TOP2B, which is associated with some treatment-induced adverse events (i.e. toxic effects of cancer therapy) (Murphy *et al.*, 2017). The ability to identify key regions in unstructured domains may support the design of new, safer therapeutics for cancer and other diseases (Biesaga *et al.*, 2021; Murphy *et al.*, 2017).

## 5 Conclusions

The *PSICalc* approach and corresponding software tool represent a novel, compact and efficient means for examining submolecular interdependencies within a protein structure using MSAs. *PSICalc* will aid researchers studying proteins of known and unknown 3D structure by identifying relationships that may have structural and/or functional significance. Additional revision and updating are warranted as we continue to test this tool against various protein datasets. Further studies are under way applying this tool to specific proteins such as topoisomerases.

## Software availability

The *PSICalc* package is licensed under the GNU General Public License v3 and is available for Python 3.6 and later. *PSICalc Viewer*, the graphical user interface which implements the *PSICalc* algorithm, is currently available for macOS 10.15 or newer. Both are available at https://github.com/jdeweeselab/psicalc-package. The python library is available for installation at https://pypi.org/project/psicalc/.

## Supplementary Material

vbac058_Supplementary_Data

## Data Availability

Datasets for Figures 1–4 and S5 are available in supplementary data. Datasets for Figure S2 and Table S1 are available upon request to the corresponding author.
