# Supplementary material for: PSICalc: a novel approach to identifying and ranking critical non-proximal interdependencies within the overall protein structure
Source: Bioinform Adv. 2022 Aug 18;2(1):vbac058. doi: 10.1093/bioadv/vbac058 (PMC9710643; doi:10.1093/bioadv/vbac058)
Supplement: vbac058_Supplementary_Data [file vbac058_supplementary_data.zip › PSICalc SI Implementation and Methods R2 22 08 11.pdf]

SUPPORTING INFORMATION FOR:

PSICalc: A novel approach to identifying and ranking critical non-proximal interdependencies within the overall protein structure

Thomas D. Townsley, et al.

Implementation and Methods:

1. Multiple Sequence Alignments as a Data Structure

We first define an MSA as an $M \times N$ matrix with the following characteristics. All individual aligned attribute sites are contained within the set *X*, which is equivalent to all columns *N*:

(S1)

$$X = \{X_1, X_2, X_3, \dots, \quad X_N\}$$

$$\text{where for } j \in 1, \dots, N,\ X_j = \begin{pmatrix} \mathrm{x_{1,j}} \\ \cdot \\ \cdot \\ \cdot \\ \mathrm{x_{M,j}} \end{pmatrix}$$

and where an individual sequence may be represented by $x_i = (x_{i,1}, x_{i,2}, ..., x_{i,j} ..., x_{i,N})$ such that *i* is the row index and *j* is the column index.

By extension the matrix form for the entire MSA is:

(S2)

$$MSA = \begin{bmatrix} x_{1,1} & \cdots & x_{1,N} \\ \vdots & & \vdots \\ x_{M,1} & \cdots & x_{M,N} \end{bmatrix}$$

where each *X* contains the entire column vector composed of individual amino acids x_{ij}.

As a final analogue to the underlying sequence data, each column vector must have a unique integer identifier based on the location in the sequence. Throughout this work, we refer to column vectors and their associated numeric labels as the attribute *A*.

2. Filtration and Labelling Methods

Since there is strong interest in measuring shared mutual information between aligned sites within an MSA, identifying aligned attributes containing a high amount of unreliable data or feature a large amount of gaps is an important preprocessing step that increases the signal-to-noise ratio in the *PSICalc* algorithm. While software-based filtering of MSA's has been shown to

distort results in cases involving phylogenetic inference or tree reconstruction, *PSICalc's* retained labelling schemes allow for *ad hoc* rules based on substitution patterns applied to the underlying data without loss to the ontology of the original MSA (Tan, et al., 2015).

There are two primary cases for building a labeled index on the MSA: The first is when the researcher has a specific sequence they want to utilize as an annotation to the global map. As shown in Figure S1, the user may select this setting in *PSICalc* Viewer which will read the label tag of the first sequence in the MSA, and annotate the MSA with that information. If a gap is found in the first sequence, the column vector is removed as it has no relation to the sequence of interest.

	59	60	61	62	63	64	65	...	199	200	201	202	203	204	205
TOP2 YEAST/59-205	G	L	F	K	I	F	D	...	F	K	P	D	G	T	R
TOP2M DICDI/168-319	T	Y	I	P	G	L	L	...	-	K	P	D	-	E	-
TOP2 BOMMO/84-234	C	S	F	P	G	L	Y	...	F	S	P	D	-	A	-
TOP2 DROME/57-206	S	F	V	P	G	L	Y	...	F	S	P	D	-	A	-

Figure S1: First-row label mapping**.** Example of a label map showing portions of four aligned sequences with sites mapped to the first sequence.

The second approach is to choose a starting label upon which to create an index at X_1 and the other columns $(X_2, X_3, \ldots, X_N)$ are labeled sequentially.

Dealing with Gaps

Gaps create a problem when analyzing MSA content as categorical values: They introduce a '-' or '.' or some combination of the two. This symbol (also referred to as the null value ∅) mostly has no meaningful relationship to the other values in the valid amino acid symbol classes:

Amino acid symbol classes: {A, C, D, E, F, G, H, I, K, L, M, N, P, Q, R, S, T, V, W, Y, ∅}

The general assumption when measuring NMI is all input data are valid labels. It is generally assumed in cases where neither column contains any gaps that the certainty of measuring intra-site variability is increased. However, when one column contains 14 gaps and another contains 14 values of Alanine, the NMI calculation will reason that the joint observation of '-' and 'A' is significant when in reality it isn't a useful indicator of site interdependence. This means the accuracy of NMI cannot be guaranteed when gaps are present in a single column vector.

To solve this issue, the *PSICalc* algorithm nullifies joint observations of gaps within the contingency matrix. Note that it is not sufficient to simply remove gaps prior to calculating NMI: Nullification at the contingency matrix level ensures the matrix transpose properties which make mutual information essentially symmetric are preserved.

The original calculation using the unmodified Pedregosa NMI library calculation was compared with the updated *PSICalc* NMI calculation using 29 multiple sequence alignments from Pfam with a broad range including manually-selected globular, fibrous, and disordered proteins. As seen in Figure S2, the original library-based NMI calculation (upper graph) leaves a broad distribution with a high-level of noise. In contrast, the *PSICalc* NMI calculation (lower graph) leads to a more symmetric distribution.

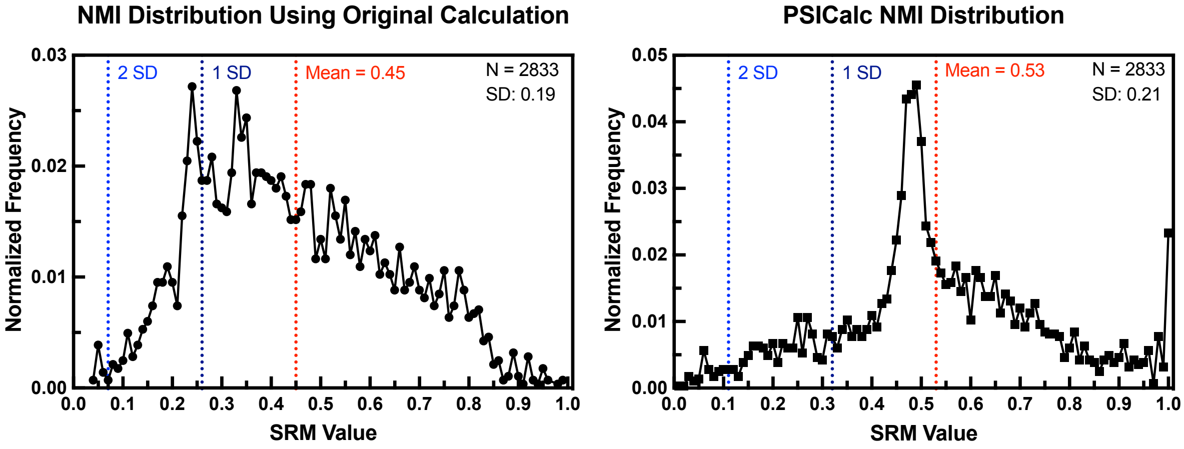

Figure S2: Statistical Redundancy Mode (SRM) Value Distribution of Pairwise Clusters Using NMI Calculation from Library Versus New NMI Calculation. Data have been normalized for comparison purposes. Data are from 29 distinct MSAs from a range of proteins from the Pfam database (Mistry, et al., 2021).

3. Numeric Substitution of Amino Acids

In addition to filtration methods described above, *PSICalc* ensures the categorical values within an MSA do not exceed the anticipated number of amino acids available in a given protein. Some MSAs obtained from public databases have shown in practice to contain erroneous data exceeding the input window of 20 standard amino acid types which can distort results in cases where distinct categorical mappings are required: For example, a lowercase letter may mean the same thing as an uppercase letter, but they will be treated as separate labels, thus modifying the mutual information score (Pedregosa, et al., 2011).

Prior to the algorithm's execution, several contingency checks are made which include (1) searching and correcting bad sequence information and (2) substituting the corrected MSA with a distinct mapping of whole numbers in a "naive" configuration, meaning 0 is retained to represent gaps in an MSA, while all standard amino acid letters are given values 1-20 and are mapped in order of appearance in the primary sequence (Figure S3).

AA Seq:	G	L	F	K	I	F	D	—	A	F	K	—	S	P
NumSub:	1	2	3	4	5	3	6	0	7	3	4	0	8	9

Figure S3: Example of numeric substitution of sequence data

Another benefit of numeric substitution is speed. Often high-performance libraries for high-level languages perform poorly when the input data do not conform to a strict numeric type. By substituting MSAs with an equivalent numeric matrix composed of typed integers, the underlying categorical values are perfectly preserved while increasing vertical scalability for large datasets and may be supported by scientific computing libraries like NumPy (Lin, et al., 2002).

4. Deriving Mutual Information from Shannon Uncertainty

The *PSICalc* approach to normalized mutual information (NMI) is based on the open source Python package by Pedregosa et al., but it has been modified under the open source BSD 3-Clause to accommodate the technical problems associated with MSAs (Pedregosa, et al., 2011).

Assuming two attributes A_1 and A_2 each representing a column vector in an MSA*,* their entropy is defined as the amount of uncertainty for the partition set $Y = \{a: a_i \in A_1\}$ and the partition set $Z = \{a: a_i \in A_2\}$ composed of the same class of *U* objects, i.e. amino acids. Therefore:

(S3)

$$P(i) = \frac{|Y_i|}{|U|}$$

is the probability that an amino acid picked randomly from *Y* falls into class Y_i and likewise with $P'(j) = |Z_j| / |U|$ (Pedgregosa, et. al., 2020, see Mathematical formulation under section 2.3.10.2). Thus we arrive at the following definition for the entropy of each partition:

(S4)

$$\mathrm{H}(Y) = -\sum_{i=1}^{|Y|} P(i)\, log_2(P(i))$$

$$\mathrm{H}(Z) = -\sum_{j=1}^{|Z|} P'(j)\, log_2(P'(j))$$

In the case of *PSICalc*, a class Y_i or Z_j is defined as a symbol from the state space of the 21 valid amino acid classes of which the column vectors are composed. For discrete random variables as in this case, the Mutual Information is defined as (Pedregosa, et al., 2020):

(S5)

$$\mathrm{I}(Y;Z) = \sum_{i=1}^{|Y|}\sum_{j=1}^{|Z|} P(i,j)\, log\left(\frac{P(i,j)}{P(i)P'(j)}\right)$$

5. *PSICalc* Algorithm

Prior to execution of the main serial algorithm, *PSICalc* discovers the strongest pairwise clusters in the MSA as a heuristic during Phase 1 (Figure S4). Doing so may provide a shortcut to obtaining the optimal early clusters to which later attribute sites are appended.

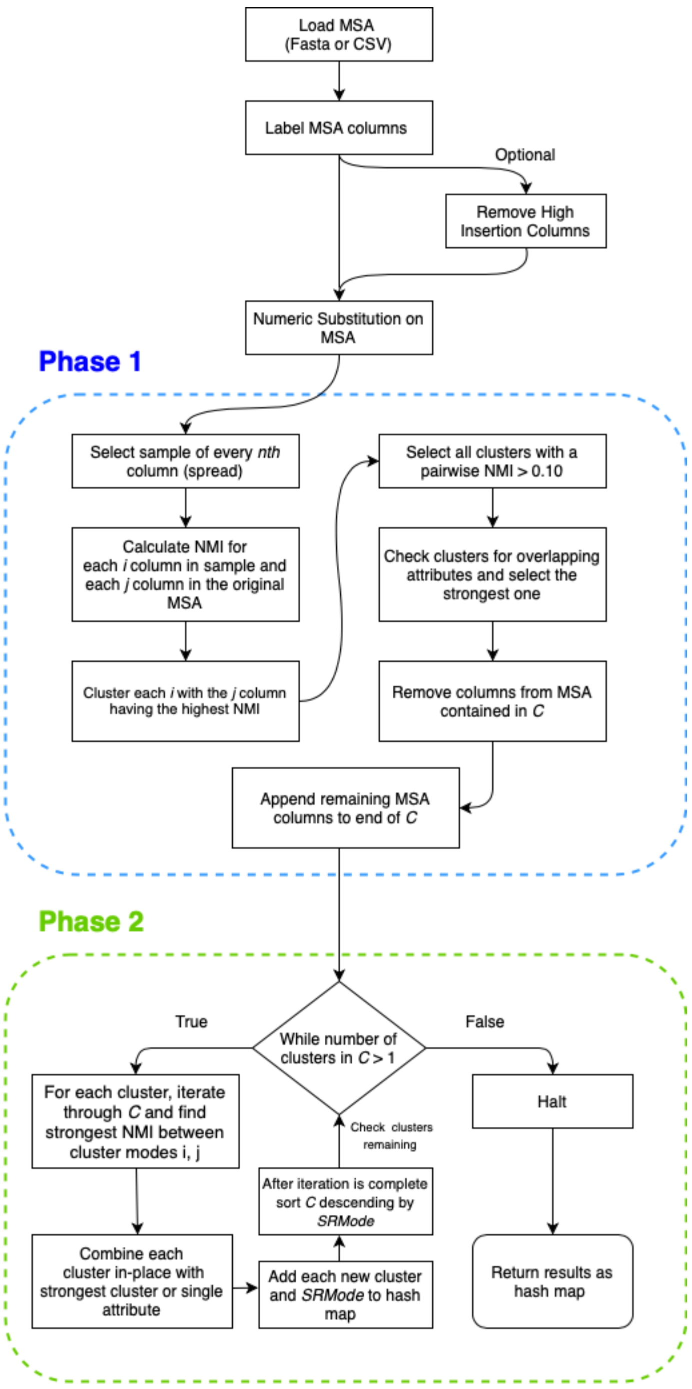

Figure S4: A high level overview of the algorithm.

5.1 Sampling the MSA

Phase 1 begins by taking a uniform sampling of every *n*-th element of the $M \times N$ data matrix along columns *N* to produce a subset MSA. If the sample spread chosen is 3 for example, then every third column of the MSA will be selected. Repeated tests on sample MSA's from Pfam show a uniform selection of every other column in *N* (setting "2" as the sample spread) is likely ideal for most cases, as it increases the number of strong pairwise clusters initially discovered. There are cases however, where increasing the spread decreases the chances of solely clustering around local contacts, which typically are characterized by a high degree of conservation. However, PSICalc does not automatically filter out clusters that are proximal in sequence, as these may have meaningful associations. Importantly, PSICalc outputs do not tend to be overwhelmed by sequence proximal clusters even with a spread of *N* = 2. 20

5.2 Selection of Clusters

Each aligned attribute column from the sample set is independently paired with the strongest found NMI value in the MSA data matrix. Here we emphasize the independence of each attribute of the sample subset in choosing the highest found attribute pair as more than one sample attribute may pair itself with the same attribute in the MSA. This results in a pairwise cluster intersecting values with another pairwise cluster. High SRM pairwise clusters with intersecting attributes will likely form a strong third order cluster.

In practice, this is a common occurrence. A common example of locality: site 3 from the sample chooses site 4 to form a cluster while site 5 from the sample also chooses 4. It is obvious then that 3, 4, and 5 are strongly related. In the case of clusters with overlapping attributes, they are compared according to their SRM values and the strongest one is retained from Phase 1 with the likelihood of the weaker local sites forming a third or fourth order cluster during the early stages of Phase 2. Once each attribute from the sample has formed a pairwise cluster and checked for overlap, the results are sorted descending by SRM value and all clusters with a value > 0.10 are retained for the final step of the Phase 1 heuristic.

5.3 Heuristic Output

Any attributes which were not clustered during Phase 1 must necessarily be retained for Phase 2 of the algorithm. To do this, we return to the original MSA data matrix and remove all columns found in the sorted ranked list. Doing so produces two distinct halves of the original MSA comprising disjoint sets of labelled attributes. All remaining attributes are added to the end of the sorted ranked list to form the Phase 1 output.

Formally the Phase 1 output is defined as the set of clusters and attributes paired with their respective SRM values:

(S6)

$$C_k = \{(s_1, SRM) \ldots (s_i, SRM),\ (A_{i+1}, 0) \ldots\ (A_n, 0)\}$$

Where *s* is a cluster obtained in the sorted ranked phase (Phase 1) and *A* is a single attribute from the MSA data matrix. Since single attributes cannot have an SRM value, they are valued at 0 to conform with the topology of the clusters.

5.4 Sequential Clustering

Phase 2 of the algorithm is the clustering phase. It is important to note that this phase, the inspiration of which was drawn from the *k*-modes algorithm, is non-random and *sequential*. This means Phase 2 is not parallelized for operation on *C* as those operations must happen in a specific order. It also means given the same exact set of input arguments, the *PSICalc* algorithm will produce the same output. The implementation therefore differs to the *k*-modes algorithm which used random selection and thus could not guarantee precisely the same set of results each time (Durston, et al., 2012; Huang, 1998).

Phase 2 begins with the highest ranked cluster at the front of the list iterating through all other clusters and single attributes to choose the cluster or attribute with the highest NMI value when predicted against the ranked cluster mode. The same process happens for all clusters in the list until the end is reached, after which the list is resorted in descending order by highest SRM value. The loop continues until all clusters consolidate together into a final single cluster, or there are only two clusters remaining. Results are then provided giving the SRM value, cluster elements, and iteration discovered for each entry in the hash table. *PSICalc Viewer* also provides a polytree graph view of the clusters, along with a spreadsheet and option to download a CSV file with the data.

Methods

In order to generate the data for this manuscript, we first identified the datasets of interest using the Pfam database. We downloaded the specific alignments in FASTA format selecting the option for sequence as “All upper case” and displaying Gaps as “-” (dashes). In order to number the clusters according to a particular family member in the MSA, we moved the specific sequence of interest to the first position in the alignment. This can be done by viewing the MSA in a text editor or spreadsheet viewer depending on the file type. The sequence of interest was chosen based upon using sequences with known 3D structure data in the Protein Data Bank (RCSB.org). To use first row mapping, the sequence name must include information about the first and last position numbers in the sequence. For datasets downloaded from Pfam, this is typically denoted as “Protein Name/10-159” where 10-159 represent the first and last amino acid position numbers in the sequence for that protein. Then, we loaded the dataset into the *PSICalc* Viewer and selected whether to number the locations in the sequence by a fixed value or to map to a particular sequence. This mapping facilitates a direct comparison between the discovered clusters in *PSICalc Viewer* with the site numbers in the 3D model. If no model is available, the researcher can choose to set the value to 1 or to map to the first sequence in the alignment. Labeling based upon the first sequence was used to provide numbering for the TOP2A dataset from the manuscript. All datasets are available upon request.

Next, we removed insertion columns in the MSA by setting a percentage value in *PSICalc Viewer* to a value that results in an MSA with the same approximate number of columns as the generally estimated length of the protein. Another method is to adjust the slider for the "percentage of non-insertion data that must be present" until the sequence for which one has a 3D model, exactly matches the same sequence located in the MSA, allowing for the possibility that some MSA's do not start at the same point as the sequence for the solved structure. A reasonable starting point is 15% and then adjust from there. This method was used for ubiquitin, and the final percentage reached was 65%. For HATPase, the final value was 15%. For the rest of the datasets analyzed in Table S1, the percentage was left at 5% since these sequences were not being analyzed further for structural correlation. For the TOP2A dataset, 35% was used because it allowed for the full length of human TOP2A to be mapped.

If one does not have a solved structure for the protein under investigation and does not even know what its essential length is, then start at 15% and adjust up or down (more likely down) until the number of columns remains stable. These can be inferred to constitute the essential sites in that protein or domain family. After this adjustment is made, the number for the actual location of the first column in the MSA may need to be re-set to the appropriate value as discussed above, unless mapping to the first sequence in the alignment. At this point 'Export MSA' can be selected to output the revised MSA.

Within *PSICalc Viewer*, the 'Spread' determines the points along the sequence that will be sampled at the beginning of the program. A spread of 2 will sample every second site/column in the MSA. Because one site may have strong interdependencies with several other sites, there may be several pairwise clusters that will be amalgamated into a higher order cluster. It is advantageous, therefore, for a thorough analysis of the protein family or domain, to run *PSICalc Viewer* at different 'Spread' settings to get a more detailed picture of the clusters and observe the effect on the cluster tree. The cluster data can be exported to a CSV file and the top clusters chosen, rated by SRM values. For the TOP2A dataset, the spread was set to 1 to obtain the data in the manuscript.

While this method does not require massive datasets, it is important for to consider that a larger sample size may tend to have more meaningful data. Having preferably 100 or more unique sequences will help strengthen the analysis and make the results more significant.

Data from 35 protein families:

		2nd Order		3rd Order		4th Order		5th Order	
Protein, Domain, or Family Name (Pfam ID)	Structure Type	Top Cluster	SRM Value	Top Cluster	SRM Value	Top Cluster	SRM Value	Top Cluster	SRM Value
Alpha-amylase (PF00128)	Globular, enzyme	18, 471	1.000	265, 270, 453	0.588	265, 268, 270, 453	0.423	265, 268, 270, 453, 456	0.328
B Lactamase2 (PF13354)	Globular, enzyme	1, 7	0.233	1, 3, 7	0.155	1, 3, 6, 7	0.112	1, 3, 5, 7, 9	0.088
BCDHK (PF10436)	Globular, enzyme	81, 315	1.000	133, 155, 181	0.664	81, 154, 192, 315	0.494	81, 154, 156, 192, 315	0.393
Chorismate Synthase (PF01264)	Globular, enzyme	84, 106	0.978	84, 93, 106	0.650	49, 87, 137, 376	0.450	49, 87, 137, 256, 376	0.347
Collagen (PF01391)	Fibrous	12, 55	1.000	2, 12, 26	0.357	1, 2, 12, 26	0.19	1, 2, 12, 26, 28	0.119
Cytochrome P460 (PF16694)	Globular, enzyme	12, 148	1.000	40, 42, 161	0.667	40, 42, 80, 161	0.500	40, 42, 80, 82, 161	0.400
Dehydrin (PF00257)	IDP/IDPD	8, 189	1.000	8, 21, 25	0.667	8, 21, 25, 28	0.500	8, 21, 25, 28, 40	0.400
DNA Pol 3 beta N-term Domain (PF00712)	Globular, enzyme	3, 73	0.902	3, 73, 109	0.577	2, 3, 4, 73	0.424	1, 69, 74, 79, 116	0.306
Fibrinogen alpha/beta chain (PF08702)	Fibrous	58, 134	1.000	58, 59, 134	0.667	58, 59, 73, 134	0.500	58, 59, 73, 77, 134	0.400
Globin (PF00042)	Globular	50, 107	0.775	50, 53, 107	0.477	2, 3, 4, 15	0.288	48, 50, 53, 84, 107	0.240
Glutathione S-Transferase N-term (PF02798)	Globular, enzyme	12, 81	1.000	12, 64, 79	0.572	12, 64, 79, 81	0.333	12, 17, 64, 79, 81	0.300
HATPase (PF02518)	Globular, ATPase	74, 119	0.779	74, 119, 125	0.489	74, 112, 113, 119	0.345	74, 112, 113, 119, 122	0.259
Histinde Kinase (PF07730)	Globular, enzyme	1, 65	1.000	29, 30, 71	0.667	29, 30, 53, 71	0.500	29, 30, 49, 53, 71	0.400
HIV Tat (PF00539)	IDP/IDPD	48, 49	0.884	19, 48, 49	0.586	19, 48, 49, 51	0.433	19, 48, 49, 50, 51	0.338
HSP70 (PF00012)	Globular, ATPase	16, 905	0.564	16, 242, 905	0.368	16, 831, 893, 905	0.258	16, 242, 537, 896, 905	0.207
HSP90 (PF00183)	Globular, ATPase	24, 158	0.990	206, 516, 517	0.655	261, 420, 424, 505	0.466	24, 158, 194, 222, 351	0.359
Insulin (PF00049)	Globular	144, 154	0.761	119, 144, 154	0.466	137, 138, 146, 153	0.334	137, 138, 145, 146, 153	0.266
LDL recptor A (PF00057)	Globular	21, 42	1.000	3, 5, 23	0.667	16, 21, 38, 42	0.226	5, 16, 21, 38, 42	0.151
Mlh1 C-terminus (PF16413)	Globular, enzyme	117, 295	0.996	117, 295, 355	0.657	117, 295, 317, 355	0.488	115, 117, 295, 317, 355	0.387
Oxidoreductase NAD bind dom (PF00175)	Globular, enzyme	1, 4	0.680	1, 4, 29	0.401	67, 78, 130, 139	0.268	1, 4, 29, 66, 77	0.209
Prothymosin (PF03247)	Fibrous	11, 47	0.813	59, 60, 106	0.511	33, 34, 45, 109	0.361	33, 34, 45, 47, 109	0.294
Pup (PF05639)	IDP/IDPD	11, 70	1.000	11, 30, 70	0.667	11, 30, 43, 70	0.500	11, 30, 43, 64, 70	0.400
RecA (PF00154)	Globular, enzyme	5, 26	0.998	5, 24, 26	0.641	1, 2, 123, 226	0.388	1, 211, 219, 226, 278	0.292
Ribosomal Protein S2 (PF00318)	Globular	36, 112	0.995	36, 112, 125	0.663	36, 112, 125, 164	0.496	36, 112, 125, 164, 204	0.395
Sec Y Protein (PF00344)	Globular, transm.	279, 310	1.000	236, 324, 330	0.665	234, 267, 276, 291	0.491	234, 267, 276, 291, 376	0.389
Shikimate quinate 5-DH (PF01488)	Globular, enzyme	148, 158	0.952	115, 172, 178	0.599	129, 177, 178, 179	0.445	129, 172, 177, 178, 179	0.339
Synuclein (PF01387)	IDP/IDPD	1, 101	1.000	1, 100, 101	0.667	63, 66, 71, 135	0.403	63, 66, 71, 132, 135	0.306
Thrombin (PF09396)	Globular, enzyme	1, 16	1.000	27, 28, 37	0.511	27, 28, 37, 40	0.331	27, 28, 37, 39, 40	0.227
Topoisomerase I, Eukaryotic (PF01028)	Globular, enzyme	10, 278	0.987	10, 278, 285	0.653	10, 278, 282, 285	0.486	246, 276, 277, 281, 283	0.375
TOPRIM, metal binding domain (PF01751)	Globular	38, 121	0.944	54, 137, 138	0.613	54, 57, 137, 144	0.436	153, 159, 160, 161, 162	0.336
Transthyretin (PF00576)	Globular, enzyme	70, 109	0.989	86, 102, 129	0.643	72, 74, 77, 84	0.473	72, 74, 77, 84, 140	0.373
Trypsin (PF00089)	Globular, enzyme	284, 305	0.898	284, 285, 305	0.552	284, 285, 287, 305	0.386	284, 285, 286, 287, 305	0.296
Ubiquitin (PF00240)	Globular	1, 60	0.311	1, 60, 61	0.206	6, 31, 33, 68	0.144	1, 11, 60, 61, 62	0.108
Ub/Sumo act enzyme ub-like dom (PF14732)	Globular	55, 65	0.994	55, 65, 98	0.644	61, 65, 69, 70	0.464	37, 61, 65, 69, 70	0.363
Zinc Finger Ring 4 (PF14570)	Globular	44, 49	0.750	42, 44, 49	0.480	42, 44, 46, 49	0.353	42, 44, 46, 48, 49	0.273

Table S1: Top clusters from 2nd to 5th order of amino acid positions from 35 protein families or domains. Cluster numbers represent amino acid positions within the primary sequence of the processed MSA alignment and will not directly correspond to a particular protein within a family (see methods for discussion of numbering according to a specific protein family member). Included above are universal proteins, as well as globular, fibrous, transmembrane (transm.) and disordered proteins. Additional higher order clusters are observed but not reported here. Due to the method of sampling, uncertainty diminishes as cluster order increases.

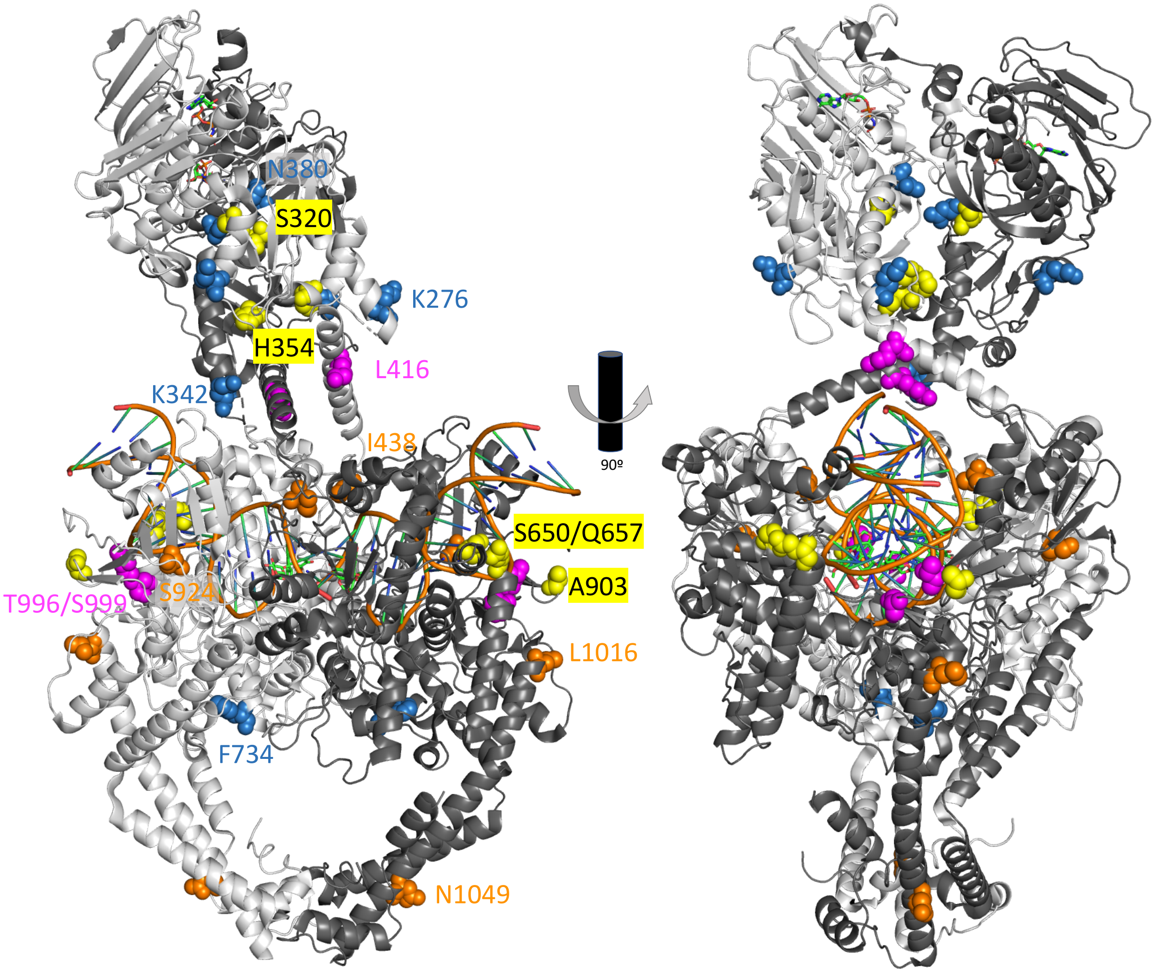

Figure S5: TOP2A homodimer is shown with color-coded clusters mapped onto the structure. Clusters are detailed in Table S2 along with functions for residues in the clusters. Images generated from PDB 6ZY7 using Pymol.

Table S2: Nonproximal, Interdomain Interdependency Examples from TOP2A

Sites	SRM Value	Notes
(276, 342, 380, 734)	0.5	K342 is part of the K-loop (K342,343,344,346) which interacts with bound DNA; K276 is on opposite face and likely interacts with DNA on the opposing side of the protomer
(416, 996, 999)	0.549231	K416, T996, S999 interact with the G-segment of DNA; K416 above and T996/S999 below
(438, 924, 1016, 1049)	0.372381	438 is at the end of the transducer; 1049 is on the lower gate; while 1016 is at the top of the lower gate upper jaw
(320, 354, 650, 657, 903)	0.372587	These residues appear to be above and below the gate segment of DNA

Coloring reflects colors in Figure S5.

References:

Durston, K.K., *et al.* Statistical discovery of site inter-dependencies in sub-molecular hierarchical protein structuring. *EURASIP J. Bioinform. Syst. Biol.* 2012;2012(8).
Huang, Z. Extensions to the k-Means Algorithm for Clustering
Large Data Sets with Categorical Values. *Data Mining and Knowledge Discovery* 1998;2:283-304.
Lin, K., May, A.C.W. and Taylor, W.R. Amino Acid Encoding Schemes from Protein Structure Alignments: Multi-dimensional Vectors to Describe Residue Types. *J. Theor. Biol.* 2002;216(3):361-365.
Mistry, J., *et al.* Pfam: The protein families database in 2021. *Nucleic Acids Res.* 2021;49(D1):D412-D419.
Pedregosa, F., *et al.* Scikit-learn: Machine Learning in Python. *J. Mach. Learn. Res.* 2011;12:2825–2830.
Pedregosa, F., *et al.* scikit-learn User Guide - 2.3 Clustering. In.; 2020.
Tan, G., *et al.* Current Methods for Automated Filtering of Multiple Sequence Alignments Frequently Worsen Single-Gene Phylogenetic Inference. *Syst. Biol.* 2015;64(5):778-791.
